# Chemotherapy-free radiotherapy combined with immune checkpoint inhibitors: a new regimen for locally advanced non-small cell lung cancer?

**DOI:** 10.20892/j.issn.2095-3941.2023.0402

**Published:** 2024-02-05

**Authors:** Lin Ma, Liufu Deng, Jianfeng Peng, Jinming Yu, Xiangjiao Meng

**Affiliations:** 1 Department of Oncology, Renmin Hospital of Wuhan University, Wuhan 430000, China; 2 Department of Radiation Oncology, Shandong Cancer Hospital and Institute, Shandong First Medical University and Shandong Academy of Medical Sciences, Jinan 250117, China; 3 School of Pharmacy, Shanghai Jiao Tong University, Shanghai 200240, China

**Keywords:** Locally advanced non-small cell lung cancer (LA-NSCLC), radiotherapy, immunotherapy, new regimen, challenges

## Abstract

Maintenance immunotherapy after concurrent chemoradiotherapy remains the standard therapeutic approach in patients with unresectable locally advanced non-small cell lung cancer (LA-NSCLC). The efficacy of pembrolizumab without chemotherapy in stage IV NSCLC has incited interest in similar approaches for LA-NSCLC. Several recent investigations involving the synergistic potential of immunotherapy combined with radiotherapy (iRT) have generated encouraging results. This review discusses the existing studies and prospective directions of chemotherapy-free iRT strategies in unresectable LA-NSCLC. Although the initial findings of chemotherapy-free iRT strategies have shown promising efficacy, we must consider the methodologic limitations of current studies and the myriad of challenges that accompany the implementation of chemotherapy-free iRT. These challenges include determining the optimal dose and fractionation, precise target volume delineation, and identification of additional suitable patient cohorts. Furthermore, the feasibility of chemotherapy-free iRT as a novel treatment modality for select patients with LA-NSCLC is contingent upon validation through randomized phase III trials.

## Introduction

Unresectable locally advanced non-small cell lung cancer (LA-NSCLC) is characterized by heterogeneity and is managed primarily by controlling local and distant micrometastases^1^. The current standard for treating LA-NSCLC without driver mutations involves consolidation with durvalumab following concurrent chemoradiotherapy (cCRT)^2,3^, as evidenced by the PACIFIC trial, in which the median PFS (mPFS) was 10.5 months. Sugemalimab consolidation post-cCRT or sequential chemoradiotherapy (sCRT) was reported to extend the PFS compared to placebo (mPFS, 10.5 months *vs*. 6.2 months)^4^. However, real world data indicate that up to one-half of patients are ineligible for CRT due to poor performance status, co-morbidities, or declining chemotherapy^5,6^, which underscores the demand for a chemotherapy-free (chemo-free) regimen in LA-NSCLC management.

The results of KEYNOTE-024 led to the endorsement of pembrolizumab as first-line treatment for advanced NSCLC patients with PD-L1 expression ≥ 50% and no driver mutations^7^. Furthermore, the CheckMate227 trial^8^ demonstrated the effectiveness of nivolumab combined with low-dose ipilimumab, which outperformed chemotherapy in first-line treatment of advanced NSCLC with improved overall survival (OS) and long-term remission. These findings bolstered the pursuit of chemo-free strategies in tumor immunotherapy and precision medicine.

The current review discusses the efficacy, practicality, and challenges of immunotherapy combined with radiotherapy (iRT) in patients with LA-NSCLC but no driver mutations, including dose optimization and radiation fractionation, improving radiotherapy techniques and multimodal image registration, and the development of novel therapeutics. We performed a systematic search in PubMed using the following terms: “NSCLC;” “LA-NSCLC;” “ICIs;” “immunotherapy;” “radiation;” and “radiotherapy.” References from relevant articles were also searched. Abstracts from American Society of Clinical Oncology (ASCO) and the European Society of Medical Oncology meetings were also included.

## Chemo-free iRT in LA-NSCLC deserves further exploration

**Figure 1:**
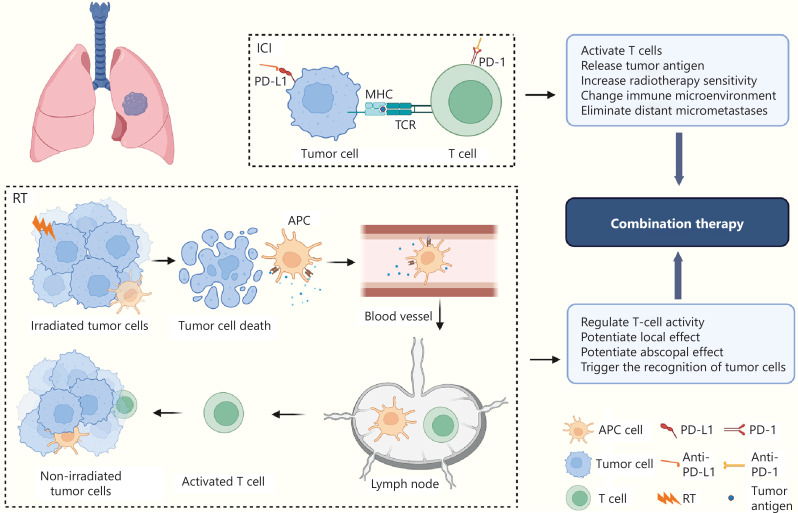
Basis of radiotherapy combined with immune checkpoint inhibitors. RT and ICI enhance each other. RT not only produces a local effect, but also releases tumor antigens, which are recognized by APCs and transported to exert abscopal effects. ICIs (mainly blocking PD-1/PD-L1) activate T cells, modify the tumor immune microenvironment, enhance RT sensitivity, and enhance the systemic anti-tumor effect of RT. RT, radiotherapy; ICIs, immune checkpoint inhibitors; APCs, antigen-presenting cells; PD-1/PD-L1, programmed cell death protein 1/programmed cell death ligand 1.

Immune checkpoint inhibitors (ICIs) significantly amplify the systemic anti-tumor effects of radiotherapy (RT)^9^. RT not only induces local tumor responses but also sparks immune-mediated anti-tumor responses through local and abscopal effects (**Figure 1**)^9^. The synergistic effect of RT combined with immunity has spurred interest in designing chemo-free clinical trials.

The anti-tumor mechanism underlying RT is multifaceted. RT directly damages tumor cells within the radiation field and provokes a systemic immune response. The systemic immune response is activated through multiple processes, such as enhancing the release and presentation of tumor antigens, promoting the initiation and activation of immune cells, and increasing the infiltration of tumor infiltrating lymphocytes^10,11^. RT activates some key immune pathways, notably the cyclic GMP-AMP synthase (cGAS)-stimulator of interferon genes (STING) pathway.

RT triggers the release of tumor cell nuclear and mitochondrial DNA into the cytosol, which is subsequently recognized by cGAS. After binding to DNA, cGAS promotes the synthesis of the second messenger, 2′3′-cyclic GMP AMP (cGAMP), which specifically activates STING, induces a significant conformational change, and migrates from the endoplasmic reticulum (ER) to the Golgi^12^. Type I interferons (IFNs), especially IFNs involved in tumor killing, are mobilized through this process, enhancing both innate and adaptive immune responses^13^. Interferon regulatory factor 3 (IRF3), and canonical and non-canonical NF-κB pathways have a crucial role in cGAS-STING-induced type I IFN expression. The canonical pathway centers around RelA-p50 and c-Rel-p50 heterodimers, while the non-canonical pathway involves RelB-p52 heterodimers^14,15^. STING oligomerizes, recruits TANK-binding kinase 1 (TBK1), activates IFN regulatory factor 3 (IRF3) to trigger IFN-β gene expression, and triggers subsequent innate immune signaling^16^. RT-induced canonical NF-κB promotes IFN-β expression and impairment of canonical NF-κB pathway diminishes the anti-tumor immunity induced by RT^17–19^. Conversely, the non-canonical NF-κB pathway exerts an opposing effect on type I IFN expression induced by the cGAS-STING signaling pathway because activated non-canonical NF-κB inhibits the recruitment of transcription factor RelA to the IFNB promoter, reducing type I IFN expression^18^.

This intricate interplay of pathways underscores the potential of RT in combination with ICIs to revolutionize the treatment landscape for cancers, such as LA-NSCLC, making a compelling case for further exploration of chemotherapy-free regimens.

### Chemo-free iRT selection based on PD-L1 showed promising outcomes

Building on KEYNOTE-024, the phase II SPRINT trial explored the iRT strategy without chemotherapy in patients with LA-NSCLC. The trial targeted patients with a PD-L1 tumor proportion score (TPS) ≥ 50%^20^. Twenty-two patients received 3 cycles of induced pembrolizumab (200 mg × 21 days) followed by risk-adapted chest RT [48 Gy/20f for tumor/nodes with a metabolic volume < 20 cc and 55 Gy/20f for tumor/nodes with a metabolic volume > 20 cc (all in 20 daily fractions)], and subsequent pembrolizumab treatments up to 12 times. The results were presented at the 2022 ASCO annual meeting showed promising outcomes with a 1-year PFS of 73%, an OS of 91%, and a mPFS of 20 months. The occurrence of grade 3 adverse events (AEs) was limited and manageable with no grade 4–5 AEs. These findings suggest that pembrolizumab combined with risk-adaptive RT achieved disease control by reducing chest irradiation with promising prognosis and manageable safety.

The SPRINT trial limited enrollment to PD-L1 expression > 50%, but approximately 30% of patients had high PD-L1 expression; therefore, a significant proportion of LA-NSCLC patients were excluded^21^. The phase II DOLPHIN study enrolled LA-NSCLC patients with PD-L1 expression > 1%, which assessed the efficacy and safety of durvalumab plus concurrent RT^22^. Patients received curative radiotherapy (60 Gy) plus durvalumab (10 mg/kg) every 2 weeks, followed by durvalumab maintenance therapy for up to 1 year. Thirty-five patients were enrolled. The 1-year PFS and objective response rate (ORR) were 72.1% (90% CI, 59.1–85.1) and 90.9% (95% CI, 75.7–98.1), respectively, which met the primary endpoint and exceeded expectations. The mPFS (investigator-assessed) at 24.1 months was superior to the mPFS in the PACIFIC trial. Based on the above results, this treatment strategy could serve as an investigational therapy in phase III clinical trials.

The mPFS in LA-NSCLC patients from other studies, such as GMSTONE-301 and the PACIFIC trial, was 10.5 and 16.8 months, respectively^23^. The KEYNOTE-799 trial, which consisted of concurrent pembrolizumab and cCRT, reported a 1-year OS of 81.2% and a PFS of 67.2%. The SPRINT and DOLPHIN trials had a better PFS than the PACIFIC trial and GMSTONE-301; the PFS was comparable to KEYNOTE-799 (**Table 1**). These results underscore the potential of iRT, particularly in PD-L1-positive LA-NSCLC patients, making this chemo-free regimen suitable for phase III clinical trials.

**Table 1:** Comparison of DOLPHIN and SPRINT clinical trials

	PACIFIC	GEMSTONE-301	KEYNOTE-799		DOLPHIN	SPRINT
			Cohort A	Cohort B		
Phase	III	III	II	II	II	II
PD-L1 (TPS)	Any level	Any level	Any level	Any level	≥ 1%	> 50%
Drug	Durvalumab	Sugemalimab	Pembrolizumab	Pembrolizumab	Durvalumab	Pembrolizumab
Radiotherapy	54–66 Gy	54–66 Gy	60 Gy	60 Gy	60 Gy	48 Gy/20f or 55 Gy/20f^a^
Intervention/treatment	Durvalumab for up to 12 months after cCRT	Sugemalimab for up to 24 months after cCRT or sCRT	Pembrolizumab plus cCRT	Pembrolizumab plus cCRT	Concurrent RT plus durvalumab for up to 12 months	3 cycles of pembrolizumab followed by RT up to 12 cycles
Primary endpoints	PFS, OS	PFS	ORR, AE	ORR, AE	1-year PFS rate	PFS
ORR	NA	NA	69.6%	70.5%	90%	NA
1-year PFS rate	55.6%	45.4%	67.2%	65.2%	72.1%	73%
1-year OS rate	83.1%	86%	81.2%	88%	NA	91%
Safety	29.9% (> grade 3)	9% (grade 3/4 AEs)	64.3% (> grade 3)	46.5% (> grade 3)	47.1% (grade 3/4 AEs)	0% (grade 4/5 AEs)

RT, radiotherapy; cCRT, concurrent chemoradiotherapy; sCRT, sequential chemoradiotherapy; OS, overall survival; PFS, progression-free survival; IDP, intrathoracic disease progression; ORR, objective response rate; IMRT, intensity-modulated radiation therapy; AE, adverse event. ^a^Hypofractionation radiotherapy with target volume based on PET-CT.

It is important to note the need for a more unified and comprehensive clinical trial design. GEMSTONE-301 and the PACIFIC trial differ in design. The lack of mandatory testing for PD-L1 expression contributed to an unknown PD-L1 expression status in 52% and 37% of patients in the GEMSTONE-301 and PACIFIC trial, respectively. The SPRINT and DOLPHIN trials were also heterogeneous in design (**Table 1**). Therefore, while the initial results were promising, standardizing trial designs, including radiation doses, fractionation, drugs, and patient selection criteria, are crucial for the accurate evaluation and advancement of iRT as a viable treatment option for patients with LA-NSCLC.

### Ongoing clinical trials of passive-type chemo-free iRT

In addition to chemo-free selection based on PD-L1 expression, the other treatment strategy is passive chemo-free. Recently, the safety and efficacy of the passive-type chemo-free regimen in elderly and frail patients with LA-NSCLC have been studied. One study investigated concurrent and consolidative durvalumab with RT (54–66 Gy/27–33f) without chemotherapy^4,24^. The results indicated a favorable safety profile for this regimen. Additionally, the phase II DUART trial (NCT04249362) evaluated the efficacy of durvalumab following a reduced dose of RT in patients unable to tolerate chemotherapy. This trial had the following two cohorts: one cohort received standard RT (54–66 Gy); and the other cohort received palliative RT (40–54 Gy). Although trial recruitment has been completed, the results are pending^25^. The combination of RT and immunotherapy is a superior treatment strategy for patients who are ineligible for chemotherapy, achieving efficacy while ensuring safety. Several clinical trials are currently underway to assess the viability of this chemo-free regimen, as summarized in **Table 2**. This development represents a significant step in expanding treatment options and personalizing care for a diverse patient population.

**Table 2:** Ongoing clinical trials of chemo-free in LA-NSCLC

NCT number	Phase	Status	Intervention model	PD-L1	Antibodies	Radiotherapy	Treatment	Primary endpoint	Secondary endpoints
NCT04013542	I	Recruiting	Single Group	Any level	Ipilimumab Nivolumab	Standard RT (60 Gy/30f)	Concurrent	Safety, Feasibility	PFS, OS, ORR, DOR
NCT04577638	II	Recruiting	Single Group	Any level	Nivolumab	IMRT (66 Gy/24f)	Concurrent	DCR	NA
NCT04249362 (DUART)	II	Active, not recruiting	Single Group	Any level	Durvalumab	Standard RT (60 Gy ± 10%); palliative RT (40–54 Gy)	Concurrent	Safety, Tolerability	PFS, OS, ORR, DOR
NCT03999710	II	Recruiting	Single Group	Any level	Durvalumab	Standard RT (60 Gy/30f)	Concurrent	2-year PFS rate	NA
NCT04929041	II/III	Recruiting	Parallel	< 1%	Pembrolizumab Ipilimumab Nivolumab	SBRT	Concurrent	PFS, OS	PFS, ORR, QoL, Safety
NCT04716946	II	Recruiting	Single Group	Any level	Durvalumab	SBRT	Concurrent	PFS	NA
NCT05451173	I/II	Not yet recruiting	Parallel	≥ 1%	Durvalumab	SBRT; Hypofractionated RT	Concurrent	MTD, AE, PFS	LCR, OS, QoL
NCT04310020	II	Recruiting	Single Group	Any level	Atezolizumab	Hypofractionated RT	Concurrent	PFS	PFS, OS, ORR
NCT03818776 (PARTICLE-D)	I	Active, not recruiting	Sequential	Any level	Durvalumab	Proton beam therapy RT (60 Gy/30f; 69 Gy/23f)	Sequential	Safety	Adverse Events, Feasibility
NCT04003246	II	Active, not recruiting	Single Group Assignment	Any level	Durvalumab	RT	Concurrent	PFS	OS, AE, DFS, Local and regional control

RT, radiotherapy; OS, overall survival; PFS, progression-free survival; DCR, disease control rate; ORR, objective response rate; DFS, disease-free survival; AE, adverse event; DOR, duration of response; MTD, maximum tolerated dose; LCR, local control rate; QoL, quality of life; EGFR, epidermal growth factor receptor; ALK, anaplastic lymphoma kinase.

## Challenge to address

### Investigating RT within a chemo-free framework

Within the realm of immunotherapy, the strategic design of RT is essential for achieving local control and mitigating risk. This necessitates a detailed exploration of various elements, including determining the optimal radiation dose, examining fractionation schedules, refining radiotherapy techniques, and accurately defining target volume.

#### Dose, fractionation, and segmentation

The DOLPHIN and SPRINT trials used conventionally fractionated RT, yet with notable differences in doses. The DOLPHIN trial administered radical RT (60 Gy/30f), whereas the SPRINT trial opted for a reduced chest RT regimen (48–55 Gy/20f). Several studies have delved into the correlation between the ideal dosing and fractionation schedules and the effectiveness of immunotherapy. Currently, while conventional dosing patterns prevail in most clinical trials, the abscopal effect, which is unique to low-dose RT, has the advantages of high-dose large-segmentation stereotactic body radiation therapy (SBRT) combined with immunotherapy over traditional RT on multiple efficacy endpoints that should be fully considered and evaluated.

##### Hypo-fractionated and SBRT

Preclinical research suggests that an optimal radiation dose per fraction when paired with systemic immunotherapy lies between 10 and 13 Gy, maximizing the anti-tumor immune response^26^. Pembrolizumab combined with high-dose RT [ORR: 24 Gy/3f, 50 Gy/4f, and 45 Gy/15f (48%, 54%, and 20%, respectively)] had higher out-of-field ORR than pembrolizumab monotherapy (ORR = 20%) based on a meta-analysis^27^. Another meta-analysis of randomized studies indicated that accelerated fractionated RT is linked with enhanced long-term survival^28^.

The NRG-LU004 phase I study evaluated the safety of accelerated and conventionally fractionated RT combined with durvalumab in 24 LA-NSCLC patients exhibiting PD-L1 expression > 50%^29^. This study involved two cohorts, as follows: cohort 1 received accelerated fractionated RT (ACRT, 60 Gy/15f) with immunotherapy; and cohort 2 received conventionally fractionated RT (CONV, 60 Gy/30f). The immunotherapy regimen involved durvalumab at a dose of 1,500 mg every 4 weeks for 1 year. Both cohorts were deemed safe and underwent further evaluation during the expansion phase. The feasibility was only attained in the ACRT cohort (85%), with at least 80% of patients in both groups receiving > 80% of the planned dose of durvalumab in the first 8 weeks. The safety of this chemo-free iRT was confirmed in this trial. At the time of analysis, 24% of patients received all 13 cycles of duvalumab. There were 4 grade 3 AEs, 1 grade 4 AE (lymphopenia), and 1 grade 5 AE (pulmonary infection, assessed as unrelated to treatment) in the ACRT (60 Gy/15f) cohort. There were 8 grade 3 AEs, 0 grade 4 AEs, and 1 grade 5 AE (respiratory failure, assessed as unrelated to treatment) in the CONV (60 Gy/30f) cohort. These findings confirm the safety and feasibility of both fractionation strategies, although the efficacy of increasing the single dose warrants further exploration in phase II and III clinical studies.

Safety is an important issue in implementing hypo-fractionated RT for patients with LA-NSCLC. With respect to safety, especially in the central type of lung cancer, the relatively large volume may affect the nearby normal structures, including the bronchial tree, heart, and esophagus. The NRG-LU004 trial indicated that the incidence of AEs is manageable. A retrospective study assessed hypo-fractionated RT and ICIs in renal cell carcinoma, melanoma, or NSCLC^30^. The incidence of any grade or grade ≥ 3 AEs was consistent with previous reports for single-agent ICI. Importantly, toxicity did not correlate with dose, fraction, or treatment sequence. A phase I trial assessed the safety of treatment-naive patients with metastatic NSCLC who received concurrent nivolumab, ipilimumab, and SBRT (45–50 Gy/3f). No dose-limiting toxicities were observed with this concurrent regimen^31^.

Organs at risk (OAR) is crucial in making dose and fraction limitation plans, especially for central lung cancer and large lung masses. Currently, a detailed investigation into the damage parameters and dose limits of OAR for hypo-fractionated RT, such as 60 Gy/15f, combined with ICI is lacking. The novel technique of a centrally dose-escalated simultaneous integrated boost (SIB) shows promise in balancing safety and efficacy. This approach safeguards adjacent OAR while enabling dose escalation to the tumor core. This technique indicates successful local tumor control in most patients with minimal pulmonary toxicity. Ten patients had local tumor control, one patient had definite progression, and one patient progressed significantly with atelectasis; grade 2 pulmonary toxicity or radiation pneumonia did not develop^32^.

A retrospective study indicated that hypo-fractionated RT reduces the risk of severe lymphopenia and improves survival in LA-NSCLC patients. The mitigation of severe lymphopenia risk may be beneficial in lessening immune suppression^33^. In microsatellite-stable colorectal cancer treated with combination of tremelimumab and durvalumab, hypo-fractionated radiation elicited greater CD8+ T-cell infiltration compared to low-dose RT^34^.

The safety of high-dose irradiation in RT is a primary concern because of the RT-related risks and the impact on immune cells, which can influence prognosis. Hypo-fractionated RT may decrease the absolute lymphocyte count and multiple activated T cell subsets in the circulation, which may affect the systemic immune response^34^. A secondary analysis of data from RTOG 0617 suggested that higher doses of radiation hinders tumor response and results in poorer outcomes^35^. Moreover, high radiation doses to the immune system are risk factors for tumor progression and mortality in patients with stage III NSCLC^36^. Therefore, RT planning must carefully weigh the potential harm to the immune system caused by irradiating organs, especially at high doses.

The combination of hypo-fractionated RT and ICIs in the treatment of LA-NSCLC patients appears promising. Comprehensive data supporting the safety and efficacy of this approach remains insufficient. Currently, hypo-fractionated RT combined with ICIs is mostly concentrated in metastatic NSCLC. Additional studies focusing on LA-NSCLC are needed to provide strong evidence of efficacy and safety.

##### Low-dose irradiation

Emerging evidence from mouse models of pancreatic cancer, orthotopic ovarian cancer, and subcutaneous lung adenocarcinoma suggests that single or multiple sessions of low-dose radiotherapy (LDRT), even at doses lower than used in conventionally fractionated radiotherapy (CFRT), elicit immunostimulatory effects and demonstrate therapeutic activity^37^. LDRT promotes differentiation of macrophages into the iNOS+/M1 phenotype, which facilitates endothelial activation, upregulates expression of TH1 chemokines, inhibits angiogenesis, and relieves immunosuppression^38^. Additionally, LDRT has been noted to upregulate T cell proliferative chemokines within tumors^39^. LDRT augments T cell infiltration and bolsters the efficacy of combined immunotherapy in an IFN-dependent manner in mouse models^40^. Tumor lesions receiving low scattered doses from high-dose irradiation of neighboring lesions have shown a more frequent response to ICI treatment^41^. A phase I study (*n* = 8, ORR = 12.5%) assessed the combination of LDRT, low-dose cyclophosphamide, and ICIs in patients with immune-desert tumors. This therapeutic combination reversed immune desertification tumors by inducing T-cell infiltration, which is predominantly composed of Th1 CD4+ cells, thereby offering a compelling rationale for the synergistic use of LDRT and immunotherapy^40^. However, studies focusing on the combination of LDRT with immunotherapy are limited. Therefore, understanding regulation of the immune response by LDRT is an area for further investigation.

#### Advances in RT techniques

The field of RT is witnessing significant advances in techniques aimed at reducing toxicity, which is particularly vital for LA-NSCLC. Proton therapy has garnered attention for the potential to reduce the risk of toxicity. Bragg peaks produced by proton and heavy ion treatment significantly reduce the radiation beam that accompanies normal tissue. This characteristic enhances the benefit-to-risk ratio, making proton therapy an appealing option.

Compared with intensity-modulated (photon) radiotherapy (IMRT), passive scattered proton therapy (PSPT) improved the heart and the cardiac radiation dose in the PSPT group was significantly lower than the intensity-modulated proton therapy (IMPT) group (*P* = 0.002)^42^. Nevertheless, another study indicated that IMPT protected the lungs, heart, spinal cord, and esophagus by reducing the dose to normal tissue compared with PSPT^43,44^. Strong evidence is needed to demonstrate the superiority of proton versus photon therapy. Further clinical studies are needed to evaluate the safety and potential survival differences between these two techniques. A trial [RTOG 1308 (NCT01993810)] is underway to expand the therapeutic window and increase the radiation dose of proton RT. The combination of durvalumab and proton beam therapy (accelerated fractionated RT) in patients with non-PD-L1 selective LA-NSCLC is ongoing [CASE1518 (NCT03818776)]. FLASH RT, with an ultrahigh dose rate, is one of the most promising breakthroughs in radiation therapy^45^. Diffenderfer et al. developed a novel RT apparatus that utilized double-scattered protons guided by computed tomography (CT) for “FLASH” proton RT. Compared to standard proton RT, this apparatus reduced acute cell loss and late fibrosis without reducing tumor suppression^46^.

Multimodal image registration is beneficial for delineating the target volume. Notably, the possibility of using positron emission tomography (PET)-CT in RT planning is a priority for future research. PET-CT quantitatively evaluates biochemical changes *in vivo*, which is beneficial for target volume, optimization of the RT scheme, and evaluation of RT efficacy^47^. The SPRINT trial used the PET-CT-guided target volume definition. Response to pembrolizumab-induced PET predicts prognosis and the 1-year PFS was 100% and 61% for pembrolizumab-induced responders and non-responders, respectively (log-rank, *P* = 0.007)^20^. Magnetic resonance imaging (MRI) has high soft-tissue resolution, which improves the accuracy of tumor description. Fusion localization of PET-CT and MRI images may help identify metastatic lymph nodes.

Besides being much safer, radiation technology has been improving continuously, and offers more individualized treatment. Proton beam and heavy ion RT, image-guided RT, and multimodal image registration of the target volume are pivotal in this evolution.

#### Target volume

In addition to exploring new RT technology, to kill the tumor more accurately and protect the normal tissue a narrowing and precise target volume is crucial. Large volume is the key to excess toxicity. Lymphocytopenia during RT is associated with gross tumor volume (GTV) and planning target volume (PTV), and larger radiation volumes are associated with severe lymphocytopenia^48^. The necessity for external expansion of the clinical target volume (CTV) must be considered. CTV omission does not compromise failure in the subclinical region. Specifically, for patients with LA-NSCLC, CTV-omitted under PET-CT guidance does not affect efficacy and may reduce severe radiation-associated toxicity^49^. This finding was a single-center phase II trial in which enrolled patients were randomized to receive CTV-omitted under PET-CT guidance (study group) or CTV-delineated (control group) combined with synchronous chemotherapy. The incidence of radiologic respiratory events or > grade 3 esophagitis was significantly lower in the study group than the control group due to reduced exposure volume of the lungs and esophagus (11.1% *vs*. 28.9%; *P* = 0.035). Interestingly, there was no significant difference in the mPFS and OS between the study and control groups (9 *vs*.10 months; *P* = 0.597 and 31 *vs*. 26 months; *P* = 0.489, respectively).

The transformation of target volume from large-scale irradiation to visible focal RT is a key challenge in target volume exploration. Exploring the reduction of target volume to enhance safety is necessary to improve the feasibility of chemo-free regimens and lay a foundation for exploring multiple treatments for LA-NSCLC.

### New antibody

In addition to the PD-1/PD-L1 pathway, new antibodies are being developed. Numerous preclinical studies have demonstrated that the T cell immunoreceptor with immunoglobulin and the ITIM domain (TIGIT) is a promising target for cancer treatment. COAST is a phase II study that assessed the efficacy of durvalumab alone or in combination with an innovative drug as consolidation therapy for LA-NSCLC with no progression after cCRT. An interim analysis showed that combined with the anti-CD73 monoclonal antibody, oleclumab, or anti-NKG2A monoclonal antibody, monalizumab, the PFS (plus oleclumab: HR, 0.44; 95% CI, 0.26–0.75; plus monalizumab: HR, 0.42; 95% CI, 0.24–0.72) and ORR (30% *vs*. 35.5% *vs*. 17.9%) were prolonged compared to durvalumab alone^50^. In addition, LAG-3 inhibitors showed significant positive results in the treatment of melanoma (relatlimab) and squamous cell carcinoma of the head and neck (eftilagimod alpha). TIM-3 is also an emerging target in immunotherapy research, although TIM-3 antibodies are not globally approved. LAG-3, TIM-3, and TIGIT have entered phase III clinical trials. We look forward to the results of ongoing and upcoming clinical trials.

### Patient selection and biomarkers

Identifying patients unsuitable for cCRT and likely to benefit from immunotherapy is important in extending the feasibility of chemo-free treatment. Indeed, we might learn from previous studies that explore the response to and benefit from immunotherapy. Previous studies on RT combined with immunotherapy have mainly focused on PD-L1 expression, the TMB, and microsatellite instability (MSI).

Currently, biomarker selection trials on chemo-free iRT focus on patients who are PD-L1-positive. The SPRINT trial enrolled patients with PD-L1 expression > 50%, while the DOLPHIN trial included patients with PD-L1 expression ≥ 1%. Several previous clinical trials showed that advanced NSCLC patients with high tumor cell PD-L1 expression tend to respond better to ICI and have longer survival^51,52^. Nevertheless, PD-L1 expression alone is not a perfect biomarker. There are many variables in tumor sampling, detection, and evaluation, and there is considerable heterogeneity in the selection of samples in time and space.

TMB, determined primarily by next-generation sequencing, is the total number of somatic mutations in the entire tumor genome, which is an emerging biomarker for predicting the prognosis of ICI. Pembrolizumab treatment is approved by the U.S. FDA for a TMB ≥ 10 mutations/Mb^53^; however, there is no consensus on the size and measurement methods of gene panels^54^. Several studies have shown that dismatch repair deficiency (dMMR) and MSI predict the ICI response^55,56^. Notably, TMB, dMMR, and MSI share similar limitations in detection, clinical application, balancing repeated sampling, and dynamic monitoring.

Circulating tumor DNA (ctDNA) analysis promotes personalization treatment. Patterns of ctDNA response during early ICI therapy identify patients who respond to treatment^57^. Moreover, dynamic changes in ctDNA are associated with clinical outcomes in LA-NSCLC. Patients with negative ctDNA collected 1 month after CRT/RT have a better PFS and OS than patients with positive ctDNA detection, and 1 month after CRT/RT may be the best time point to detect ctDNA for predicting prognosis^58^. Monitoring ctDNA dynamics can monitor the efficacy in real time, select individualized treatment plans, adjust treatment strategies in time, and avoid unnecessary adverse events.

Tumor infiltrating lymphocytes (TILs) exert strong anti-tumor immune responses. The PD-1^+^ stem-like exhausted T cell subset mediates the long-term response to anti-PD-1 therapy and is associated with immune efficacy^59^. The increase in CD8^+^ TIL density after RT is also associated with improved clinical outcome^60^. The immune regulatory network of the tumor microenvironment (TME) is large and complex, and includes immune cells, cytokines, and exosomes. Recently, the PD-1 expression balance of CD8^+^ T and Treg cells has been shown to predict clinical responses to PD-1 blockade therapy^52^. Unlike the unique localization of PD-L1 in tumor cells, the complexity of histologic biopsies limits the direct assessment of PD-1^+^ TIL.

For high-risk patients unsuitable for cCRT, safety is the first priority. A comprehensive evaluation system is needed to identify patients who are not candidates for cCRT. Immunologic, such as cytokine levels and HLA type, radiomic parameters, Eastern Cooperative Oncology Group (ECOG) performance status, and peripheral blood indicators, may assist in identifying high-risk patients. Overlapping toxicities may arise from combined immunotherapy, RT, and systemic therapy, with immunologic- and RT-induced pneumonia requiring monitoring. Currently, there is a lack of clinical and radiologic tools to differentiate between immunologic- and RT-related pneumonia, highlighting the need for improvements in radiomics for better management. Identifying patient subgroups at higher risk and developing radiomic tools to manage immune/radiation-associated AE is crucial for treatment of LA-NSCLC patients.

Although there are multiple levels of biomarkers that provide direction, we are still far from accurate screening of patients most likely to benefit from chemo-free iRT. Accurate and comprehensive prediction of treatment response should not be limited to a single biomarker to identify the population with potential benefit. Multi-omics integration of patient characteristics, pathology, genome, and imaging will help guide personalized treatment. This is a critical area in need of further exploration. We look forward to future work focusing on this aspect and a detailed investigation.

### Future directions

A thorough and comprehensive understanding of the mechanism underlying the interaction between RT and immunotherapy is pivotal for accurately identifying appropriate LA-NSCLC patients. Conventional target volume, fractionation, and dose did not pay in-depth attention to the potential RT immune stimulation. To explore different radiotherapy doses, fractionation schemes and target volumes to maximize the immune synergistic effect of RT are needed. Randomized clinical trials are necessary to provide reliable clinical data to determine the optimal combination of immunotherapy and RT. To carry out randomized clinical trials, it is necessary to establish a relatively unified dose and target volume design. Previously published randomized trials, such as SPRINT, DOLPHIN, and NRG-LU004, reflect inconsistencies in study design, choice of ICIs, end-point selection, and patient enrollment criteria. Chemo-free immunotherapy combined with RT may only be suitable for some patients. Efficacy evaluation and biomarker exploration are also valuable.

## Conclusions

**Figure 2:**
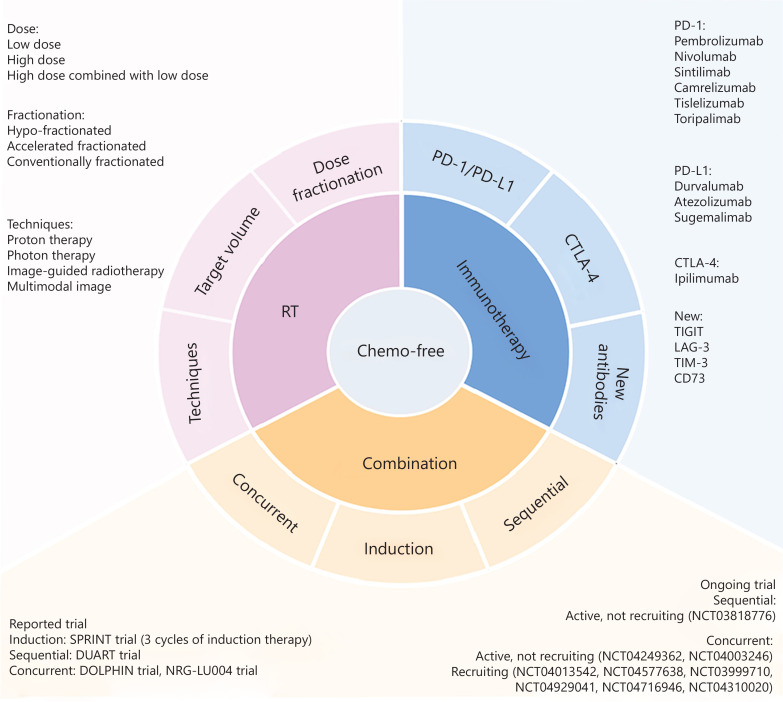
Exploration direction of chemotherapy-free model. Chemo-free, chemotherapy-free; RT, radiotherapy; ICI, immune checkpoint inhibitor.

The emergence of precision medicine has facilitated the development of chemo-free strategies that rely on immunotherapy, which has demonstrated efficacy in advanced stage IV NSCLC. This review summarized chemo-free clinical trials in patients with unresectable LA-NSCLC, including passive chemo-free modes for chemotherapy-intolerant patients and biomarker-based chemo-free models. Although current studies show favorable safety and feasibility, several aspects need exploration, including dose and fractionation schedules, advanced RT techniques, new immune antibodies, and combination therapy (**Figure 2**). Such studies are currently ongoing, phase III clinical trials are warranted (**Table 2**), and future studies are expected to enrich combination therapies, leading to more options for patients with LA-NSCLC. The development of this chemo-free iRT strategy is expected to lead to significant advances in the treatment of LA-NSCLC, highlighting a shift towards more targeted, less toxic, and potentially more effective treatment modalities, thus reflecting a new era in the fight against lung cancer.
